# NONMMUT140591.1 may serve as a ceRNA to regulate Gata5 in UT-B knockout-induced cardiac conduction block

**DOI:** 10.1515/biol-2021-0106

**Published:** 2021-11-27

**Authors:** Xuejiao Lv, Yuxin Sun, Wenxi Tan, Yang Liu, Naiyan Wen, Shuang Fu, Lanying Yu, Tiantian Liu, Xiaocui Qi, Nanqi Shu, Yanwei Du, Wenfeng Zhang, Yan Meng

**Affiliations:** Department of Respiratory Medicine and Pathophysiology, Jilin University, No. 218, Ziqiang Road, Nanguan District, Changchun, 130041 Jilin, China; Department of Otolaryngology, Jilin University, Changchun, Jilin, 130021, China; Department of Nursing, Changchun University of Chinese Medicine, Changchun, Jilin, 130117, China; Department of Pathology and Pathophysiology, Changchun University of Chinese Medicine, Changchun, Jilin, 130117, China; Department of Prescriptions, Changchun University of Chinese Medicine, Changchun, Jilin, 130117, China

**Keywords:** cardiac conduction disease, lncRNA, mRNA, co-expression, ceRNA

## Abstract

We intended to explore the potential molecular mechanisms underlying the cardiac conduction block inducted by urea transporter (UT)-B deletion at the transcriptome level. The heart tissues were harvested from UT-B null mice and age-matched wild-type mice for lncRNA sequencing analysis. Based on the sequencing data, the differentially expressed mRNAs (DEMs) and lncRNAs (DELs) between UT-B knockout and control groups were identified, followed by function analysis and mRNA–lncRNA co-expression analysis. The miRNAs were predicted, and then the competing endogenous RNA (ceRNA) network was constructed. UT-B deletion results in the aberrant expression of 588 lncRNAs and 194 mRNAs. These DEMs were significantly enriched in the inflammation-related pathway. A lncRNA–mRNA co-expression network and a ceRNA network were constructed on the basis of the DEMs and DELs. The complement 7 (C7)–NONMMUT137216.1 co-expression pair had the highest correlation coefficient in the co-expression network. NONMMUT140591.1 had the highest degree in the ceRNA network and was involved in the ceRNA of NONMMUT140591.1-mmu-miR-298-5p-*Gata5* (GATA binding protein 5). UT-B deletion may promote cardiac conduction block via inflammatory process. The ceRNA NONMMUT140591.1-mmu-miR-298-5p-*Gata5* may be a potential molecular mechanism of UT-B knockout-induced cardiac conduction block.

## Graphical abstract

**Figure:**
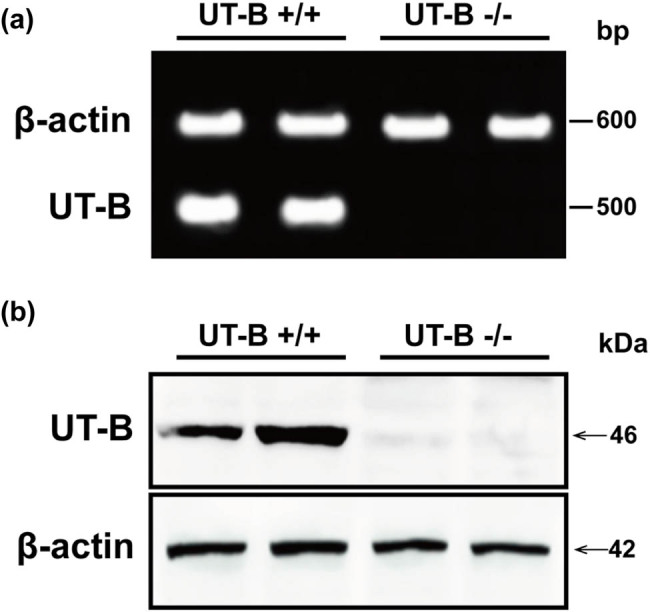

## 1 Introduction

Cardiac conduction disease, characterized by the impaired integrity of the conduction system, is a serious, life threatening disease of the heart [1,2]. An impaired conduction system can result in slowed or even blocked impulse conduction, eventually leading to life-threatening rhythm disturbances [3]. The pathogenesis of the cardiac conduction disease is diverse, which can be caused by an acquired injury such as drug toxicity or ischemia, and is associated with heart diseases and neuromuscular diseases [4]. In the last decade, some ion channels associated genes that are responsible for the inherited cardiac conduction disease have been detected, such as *KCNJ2*, *SCN5A,* and *HCN4* [5].

Urea transporters (UTs) are a family of small membrane proteins with specific permeability to urea, which include two types in mammals: UT-A and UT-B [6]. UT-A is mainly expressed in the kidney epithelial cells, while UT-B is widely distributed in the brain, heart, kidney, testis, bone marrow, urinary tract, and other tissues [7]. UT-B has a high expression in heart. Our previous study has found that UT-B deletion mice have prolonged P–R intervals from 6 to 52 weeks, indicating a delayed conduction from the atria to the ventricles [8]. Meng et al. [9] reported that the progressive heart block in UT-B null mice may be associated with the accumulated urea in cells. Urea has been considered to have negligible toxicity for a long time. Elevated blood urea in chronic renal failure is thought to have no influence on survival in patients [10]. High urea might have an important role in accelerated atherosclerosis in chronic dialysis patients [11]. Nevertheless, the role of urea accumulation in heart disease is still controversial.

In this study, the urea transporter B null mice and all urea transporters null mice provide us with proper tools to eliminate other interference factors. We have subtly controlled the concentration of urea through the knockout of the urea transporters to explore the potential molecular mechanisms underlying the cardiac conduction block induced by UT-B deletion at the transcriptome level. The RNA expression profiles of the heart tissues in UT-B knockout mice were analyzed by comparing with age-matched wild-type mice. The differentially expressed mRNAs (DEMs) and lncRNAs (DELs) between UT-B knockout and control groups were identified. Based on these DEMs and DELs, the competing endogenous RNA (ceRNA) network was constructed to explore the possible mechanism. The results may improve our understanding of the progression of cardiac conduction defect.

## 2 Materials and methods

### 2.1 Animals and tissue preparation

**Figure 1:**
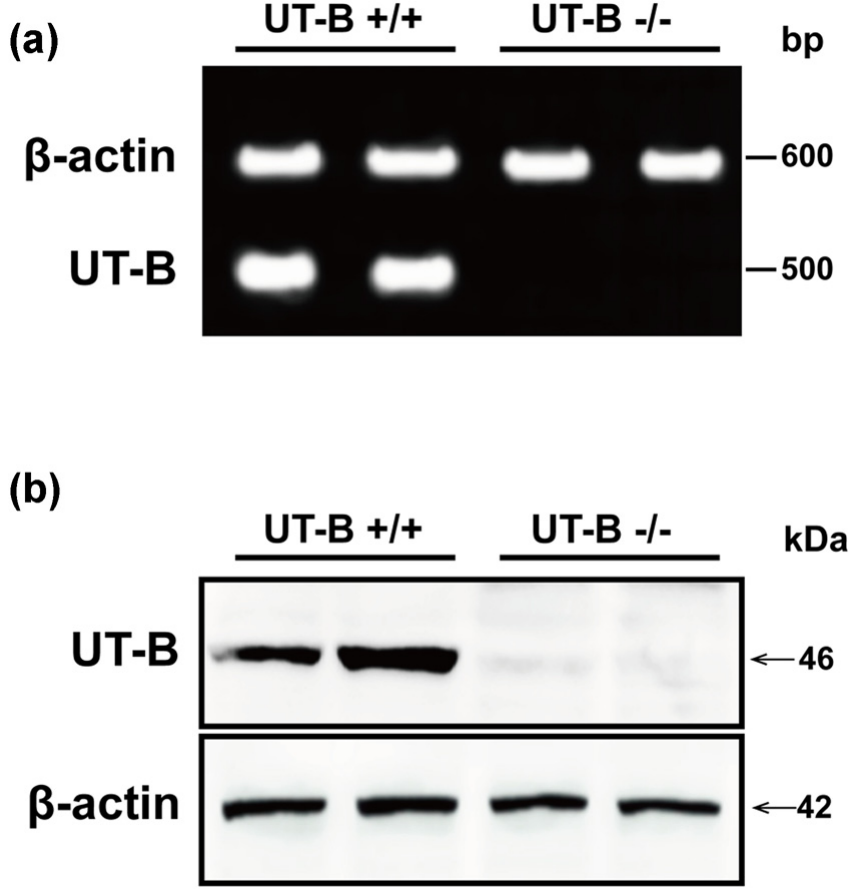
The expression of UT-B in UT-B null mice detected by reverse-transcription (RT)-PCR (a) and western blot (b).


UT-B null mice were kindly provided by the University of California, San Francisco, School of Medicine. All the animals were kept in a standard experimental animal laboratory at the animal experiment center of Jilin University. Mice were raised with plenty of food and water under a 12:12 h light:dark cycle at 22 ± 2°C. In order to obtain stable UT-B null mice, the mice were mated with wild-type mice to obtain a heterozygous offspring. Then the heterozygous offspring were mated to obtain stable UT-B knockout mice, and wild-type mice from the same litter were used as control. At 16 weeks of age, three wild and null mice were respectively selected and anesthetized with 1% pentobarbital sodium. The hearts were harvested through thoracotomy and frozen in liquid nitrogen for sequencing analysis. The expression of UT-B in the UT-B knockout mice was verified, as shown in Figure 1.

**Ethical approval:** The research related to animal use has complied with all the relevant national regulations and institutional policies for the care and use of animals, and were approved by the animal ethics committee of the Basic Medical Sciences unit of Jilin University (No. [2021]202014).

### 2.2 RNA extraction and RNA library construction for sequencing

Total RNA was extracted from heart tissue using TRIzol (Invitrogen; Carlsbad, CA, United States). The RNA quality was detected through gel electrophoresis. The concentration of RNA was detected using a NanoDrop ND-1000 spectrophotometer.

After removal of rRNA from the total RNA, the RNA samples were randomly cut into short fragments which were used to synthesize the first-strand cDNA using random hexamers. The synthesis of second-strand cDNA was performed through the mixing of first-strand cDNA with dNTPs, RNase H, and DNA polymerase I. After purification, the cDNA was degraded with uracil-*N*-glycosylase. The RNA fragments were separated using agarose gel electrophoresis, followed by amplification with polymerase chain reaction (PCR). The PCR products were sequenced on an Illumina HiSeq™ 2000 instrument (Illumina, Inc.; San Diego, CA, United States). The raw data were uploaded to the Gene Expression Omnibus (GSE168717, https://www.ncbi.nlm.nih.gov/geo/query/acc.cgi?acc=GSE168717).

### 2.3 Correlation analysis and principal component analysis (PCA) among samples

The expression level correlation between samples is an important index to test the reliability of the experiment and the rationality of sample selection. Using the cor function in R3.4.1, the Pearson correlation coefficient (*p*) between the two samples was calculated. For PCA, the prcomp function was used for data dimension reduction, and the ggfortify package was used to draw the PCA map.

### 2.4 DEMs and DELs identification

DESeq [12] software was used to conduct normalization processing on the counts of the genes of each sample (expression quantity was estimated using basemean value), and to calculate the fold change (FC). The negative binomial distribution test was used to conduct the difference significance test on reads. The differential genes (DEMs and DELs) were screened according to the FC and the results of the difference significance test. Here, the screening conditions were *p* < 0.05 and the FC >2.

### 2.5 Function and pathway enrichment analyses

After getting the differentially expressed genes, gene ontology (GO) (molecular function [MF], biological process [BP], and cellular component [CC]), and the Kyoto Encyclopedia of Genes and Genomes (KEGG) pathway enrichment analyses were performed by combining the GO [13] and KEGG [14] databases. The significance of DEMs enrichment in each GO term or pathway was calculated by the hypergeometric distribution test. The *p* value was adjusted using the Benjamini and Hochberg method. The threshold was set as count ≥3 and adjusted *p* value was <0.05.

### 2.6 Co-expression analysis of DELs and DEMs and function analysis of DELs

The Pearson correlation test was used to calculate the expression correlation between DELs and DEMs based on the gene expression data. The threshold values were set as |cor| >0.8 and *p* value <0.05. For each of the DELs, their co-expressed mRNAs were calculated, and the significance of DEMs enriched in each GO (or pathway) terms was calculated using the hypergeometric distribution test. The *p* value was adjusted using the Benjamini and Hochberg method. The threshold was set as count ≥3 and adjusted *p* value <0.05.

### 2.7 *Cis* of lncRNA and its adjacent coding gene analysis and lncRNA *trans* analysis

All the coding genes within the range of 100k in the upstream and downstream of DELs were searched, which were intersected with the genes that were significantly co-expressed with the lncRNAs (Pearson correlation calculation). These genes that were close to each other on the genome and had co-expression patterns were likely to be regulated by this lncRNA.

Based on the co-expression analysis results, the lncRNAs and mRNAs that were not on the same chromosome were screened out as candidate targets and the candidate sequences were extracted. The binding of candidate lncRNAs and genes at the nucleic acid level was predicted using RIsearch-2.0. The screening conditions were: the number of bases on which the two nucleic acid molecules interacted directly must be ≥10 and the base binding free energy ≤−50.

### 2.8 miRNA prediction and ceRNA network analysis

On the basis of the co-expression relationship of lncRNA–mRNA, the upstream miRNAs of mRNAs were predicted using the validated target module in the miRWalk2.0 [13] tool [15]. For lncRNAs in the lncRNA–mRNA co-expression relationship, we predicted the miRNA binding sites of the lncRNAs through miranda (v3.3a) [16] with parameters of score ≥140 and energy ≤−20, and obtained the miRNA–lncRNA relationship pairs. Based on the obtained lncRNA–miRNA and mRNA–miRNA relationships, the lncRNA–miRNA–mRNA relation pairs were screened, which were further screened according to the positive co-expression relationship between mRNA and lncRNA (correlation coefficient >0.95). Cytoscape (version 3.4.0) [17] was used for network building, namely the ceRNA network. The lncRNAs and mRNAs that had a positive co-expression relationship and were regulated by the same miRNA in the ceRNA network were considered as ceRNA. Finally, the node connection degree was analyzed using the Cytoscape plugin CytoNCA (version 2.1.6) [18].

## 3 Results

### 3.1 Correlation analysis and PCA among samples

**Figure 2:**
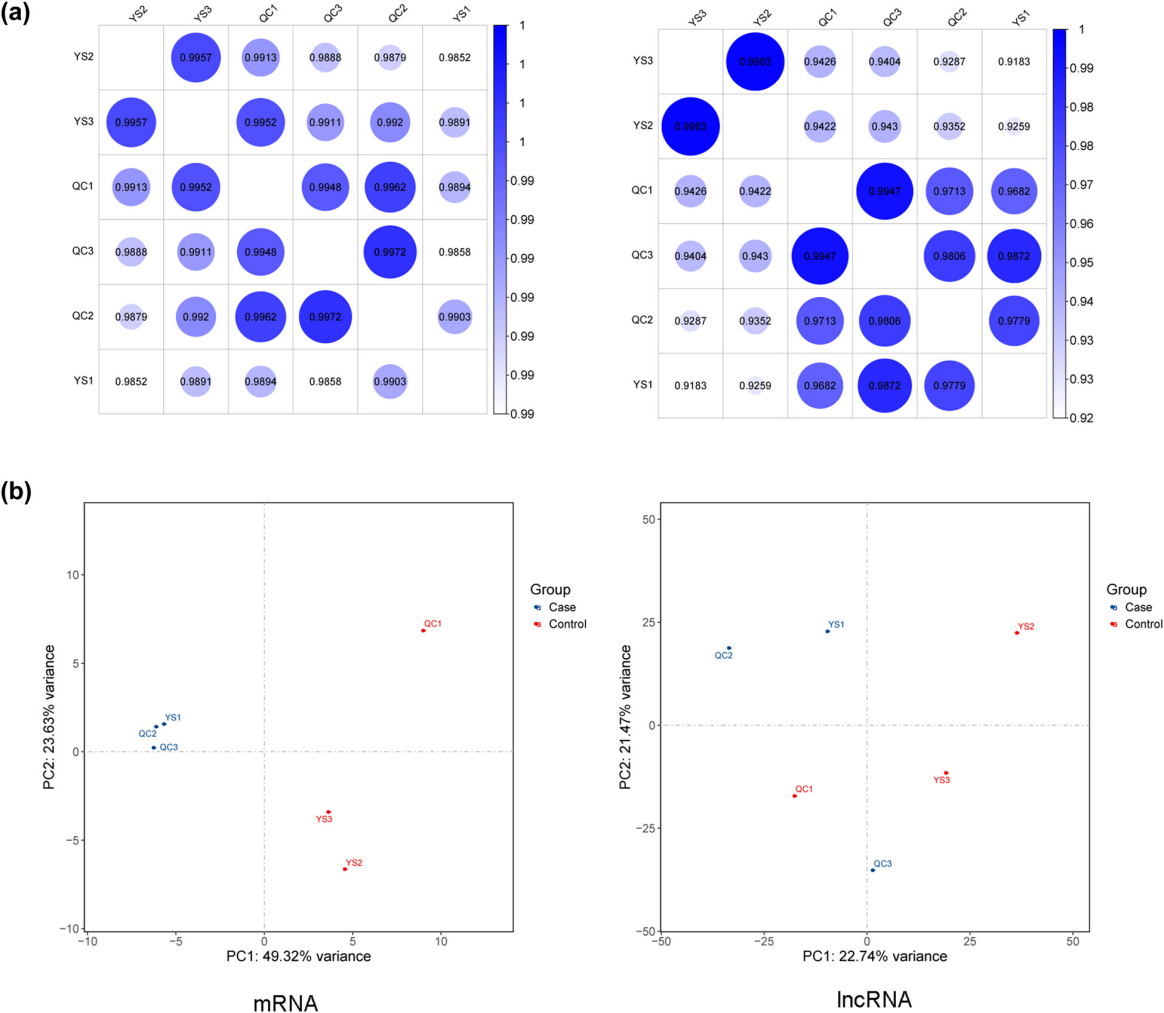
(a) Sample correlation heatmaps based on the expressive abundance of mRNA and lncRNA and (b) PCA maps for mRNA and lncRNA.

Based on the mRNA and lncRNA expression matrices of each sample, the correlation between the samples was evaluated through the Pearson correlation coefficient. The closer *p* was to 1, the higher was the similarity of expression patterns between the samples (Figure 2a). The PCA map is shown in Figure 2b, indicating that the samples in the two groups were completely separated.

### 3.2 Differential expression analysis

**Figure 3:**
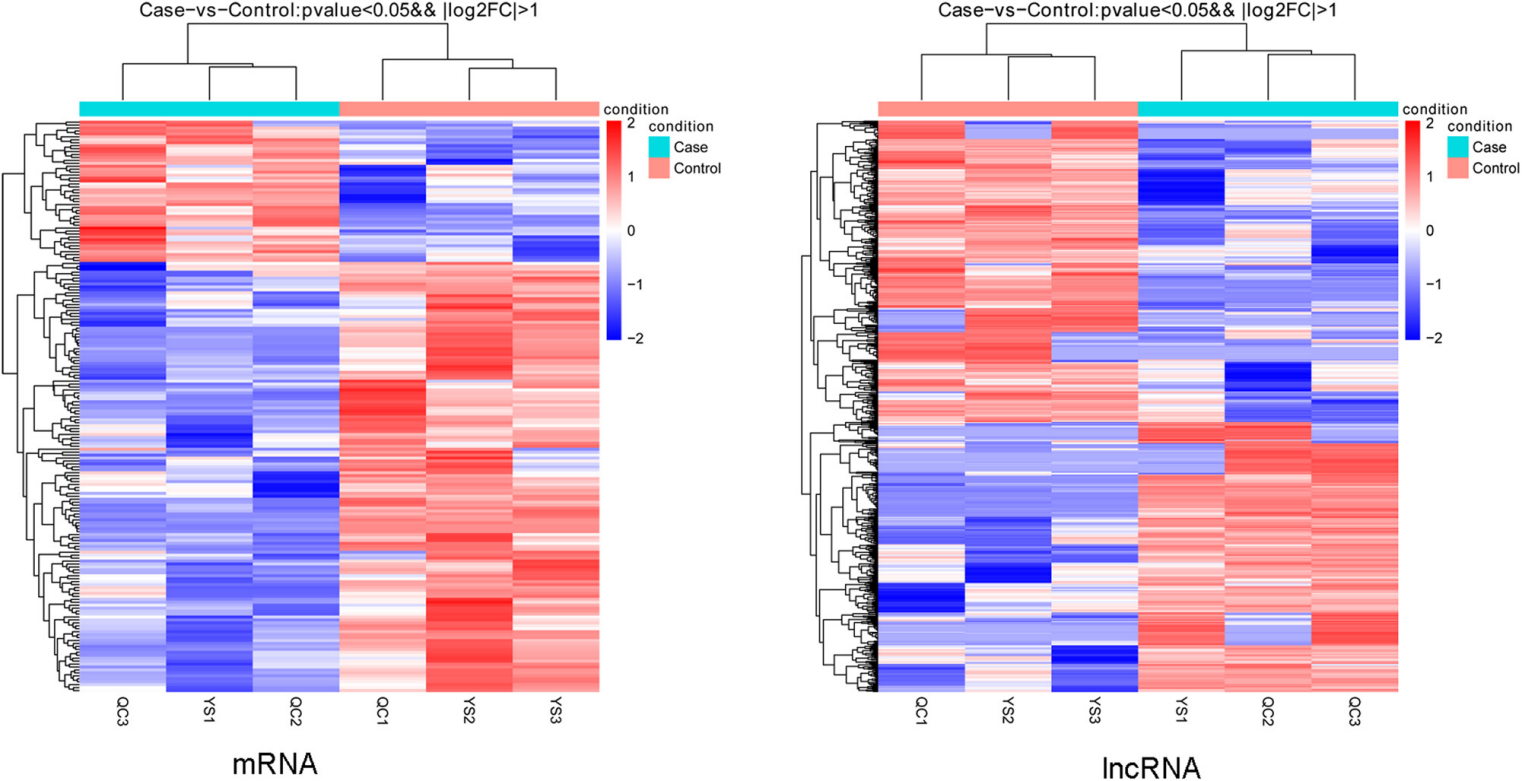
The clustering heatmaps of mRNAs and lncRNAs.

According to the thresholds of *p* value <0.05 and |log2FC| >1,588 (278 up- and 310 downregulated) DELs and 194 (48 up- and 194 downregulated) the DEMs were identified. There were a greater number of downregulated than upregulated DELs and DEMs, and the NONMMUT140591.1 was a downregulated lncRNA. Bidirectional hierarchical clustering heatmaps are shown in Figure 3, which suggested that DELs and DEMs were well distinguished between the samples from the samples in the two groups.

### 3.3 Functional enrichment analysis of up- and downregulated DEMs

**Figure 4:**
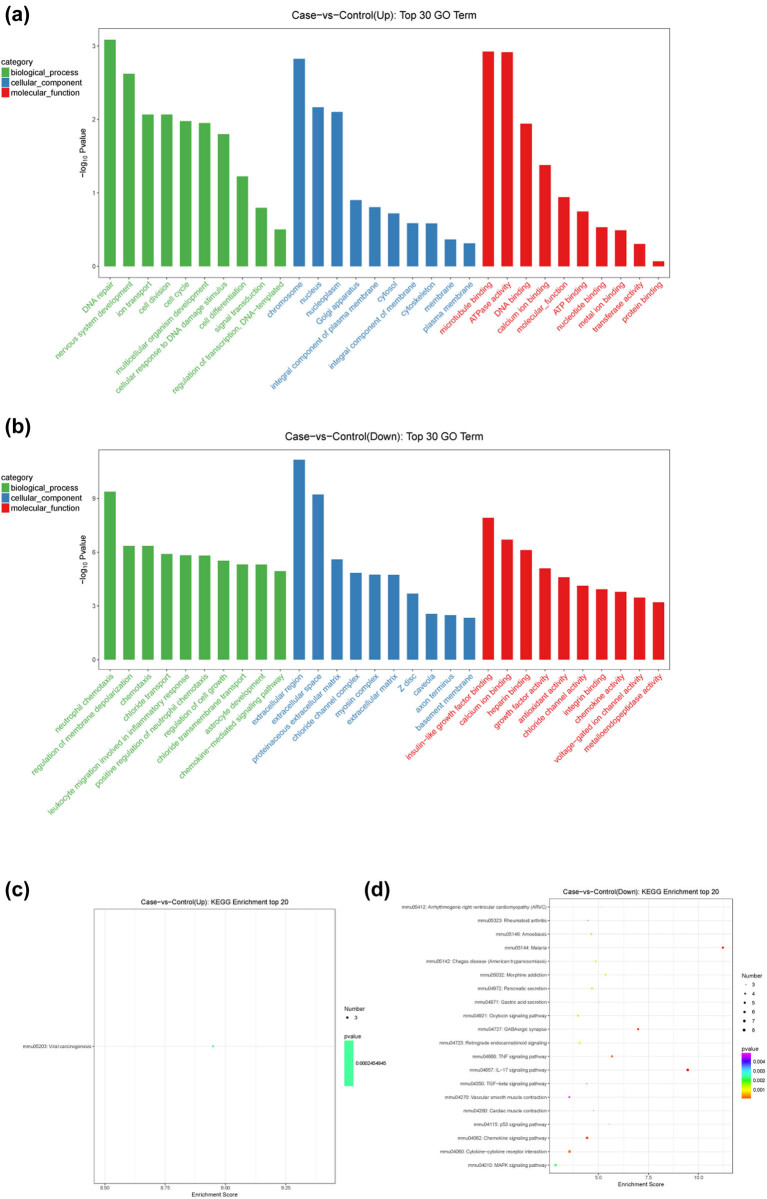
The functions (a and b) and pathways (c and d) enriched by up- and downregulated mRNAs.

The GO (BP, CC, and MF) functional enrichment analysis and the KEGG pathway enrichment analysis were performed on the up- and downregulated mRNAs, respectively. The results showed that seven BPs (such as DNA repair, nervous system development, and ion transport), three CCs (chromosome, nucleus, and nucleoplasm), three MFs (microtubule binding, ATPase activity, and DNA binding) (Figure 4a), and one KEGG pathway (viral carcinogenesis) (Figure 4c) were significantly enriched on the up-regulated mRNAs. Downregulated mRNAs were significantly enriched in 64 BPs (such as neutrophil chemotaxis, chemotaxis, and regulation of membrane depolarization), 19 CCs (such as extracellular region and extracellular space), 21 MFs (insulin-like growth factor binding and calcium ion binding) (Figure 4b), and 35 KEGG pathways (such as cytokine–cytokine receptor interaction, chemokine signaling pathway, interleukin (IL)-17 signaling pathway, and mitogen‑activated protein kinase signaling pathway) (Figure 4d).

### 3.4 lncRNA–mRNA co-expression analysis

**Figure 5:**
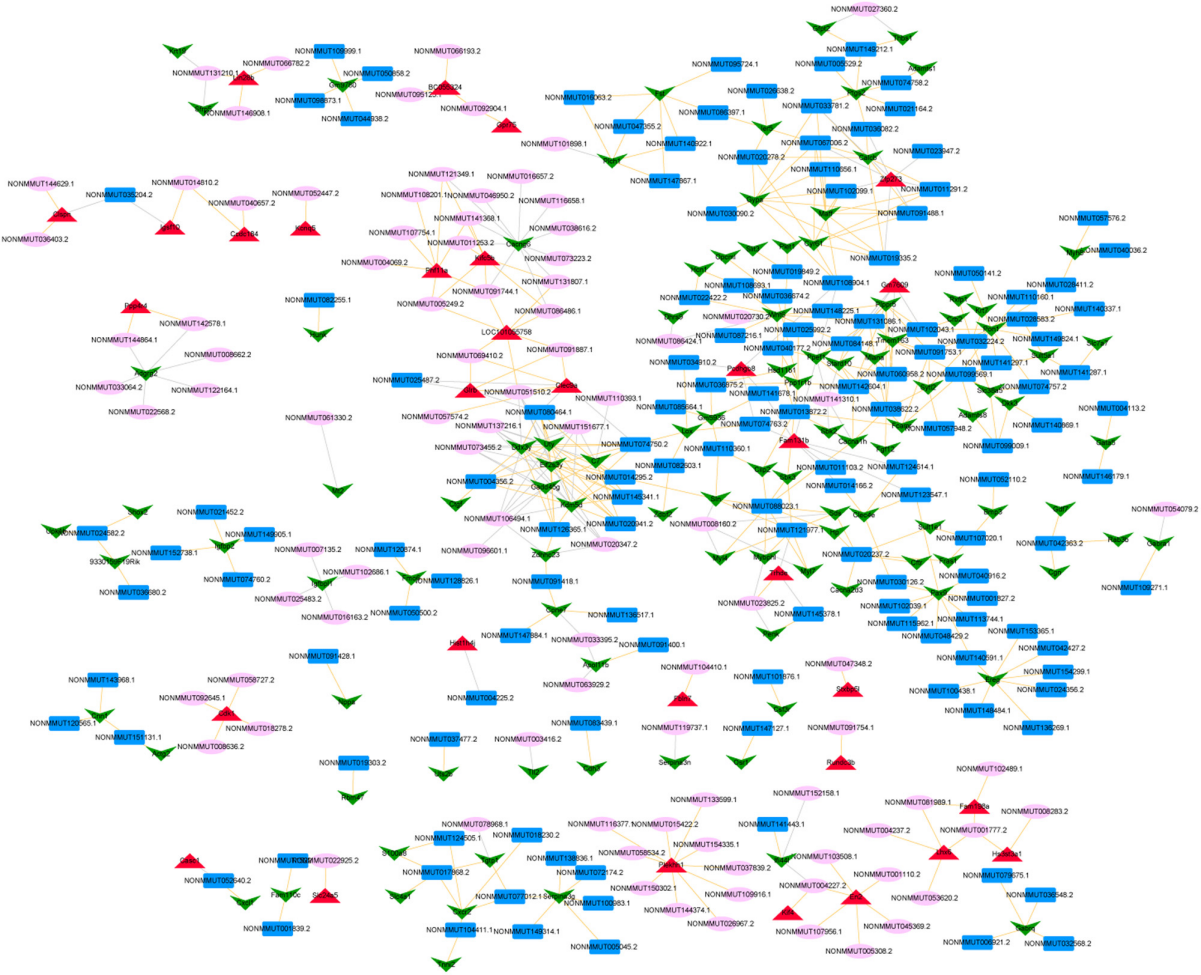
mRNA–lncRNA co-expression network (red triangle represents upregulated mRNA; the green inverted triangle represents downregulated mRNA; pink ellipse represents the upregulated lncRNA; the blue rectangle represents the downregulated lncRNA; the yellow line represents the positive co-expression relation; and the gray line represents the negative co-expression relation).

A total of 27,640 lncRNA–mRNA co-expression relation pairs, including 587 lncRNAs and 194 mRNAs, were obtained under |cor| >0.8 and *p* value <0.05. The top 500 pairs according to the *p* values were selected to construct the co-expression network (Figure 5), and the C7–NONMMUT137216.1 co-expression pair had the highest correlation coefficient in the co-expression network.

### 3.5 Function analysis of DELs

The top 10 lncRNAs, including NONMMUT040916.2, NONMMUT048938.2, NONMMUT065231.2, etc., with the largest number of GO enrichment (>5) were selected, and the top 30 of the GO terms were displayed (Supplementary file 1). For example, the positive regulation of the transforming growth factor beta production, extracellular region, and heparin binding were significantly enriched on the NONMMUT040916.2. Additionally, the top 10 lncRNAs, containing NONMMUT001827.2, NONMMUT019380.2, NONMMUT026967.2, etc., with the largest number of KEGG enrichment (no less than 2) were selected, and the top 20 of the pathways were displayed (Supplementary file 2). For example, malaria, TNF signaling pathway, and IL-17 signaling pathway were significantly enriched on the NONMMUT001827.2.

### 3.6 *Cis* and *trans* analyses

As mentioned in the method section, we found that *Kdm5d*, *Eif2s3y,* and *Uty* were regulated by NONMMUT074750.2; *Adcy1* was regulated by NONMMUT140337.1; *LOC101055758* was regulated by NONMMUT001777.2; *Ddx3y* and Uty were regulated by NONMMUT074763.2; *Cacna2d3* was regulated by NONMMUT092640.1; *Igfbp2* was regulated by NONMMUT001471.2; and *Uty* was regulated by NONMMUT074758.2.

**Figure 6:**
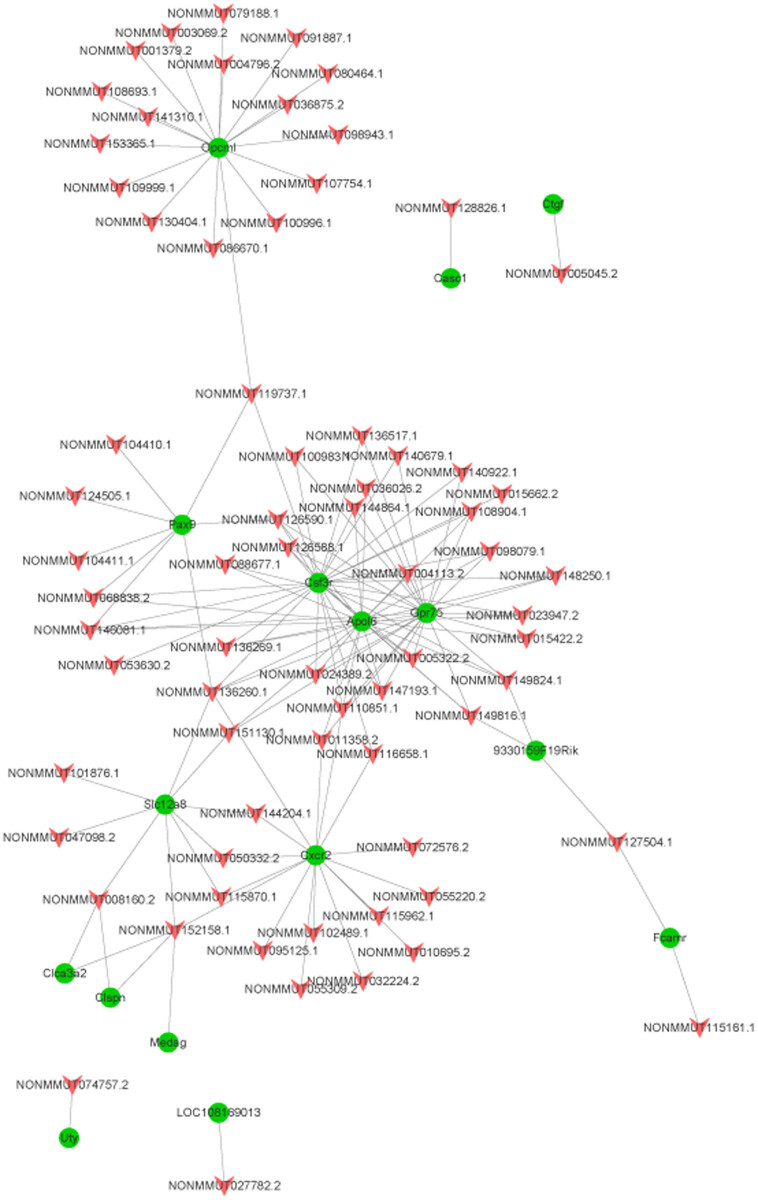
Network of lncRNA and gene *trans* analysis (red arrow nodes represent differentially expressed lncRNAs, while green circular nodes represent DEMs).

The binding free energy was ranked from small to large, and the top 200 were selected to construct the network, as shown in Figure 6. There were 16 mRNAs in this network. *Opcml*, *Pax9*, *Csf3r*, *Apol6*, *Gpr75*, *Slc12a8,* and *Cxcr2* interacted with numerous lncRNAs.

### 3.7 miRNA prediction and ceRNA network construction

**Figure 7:**
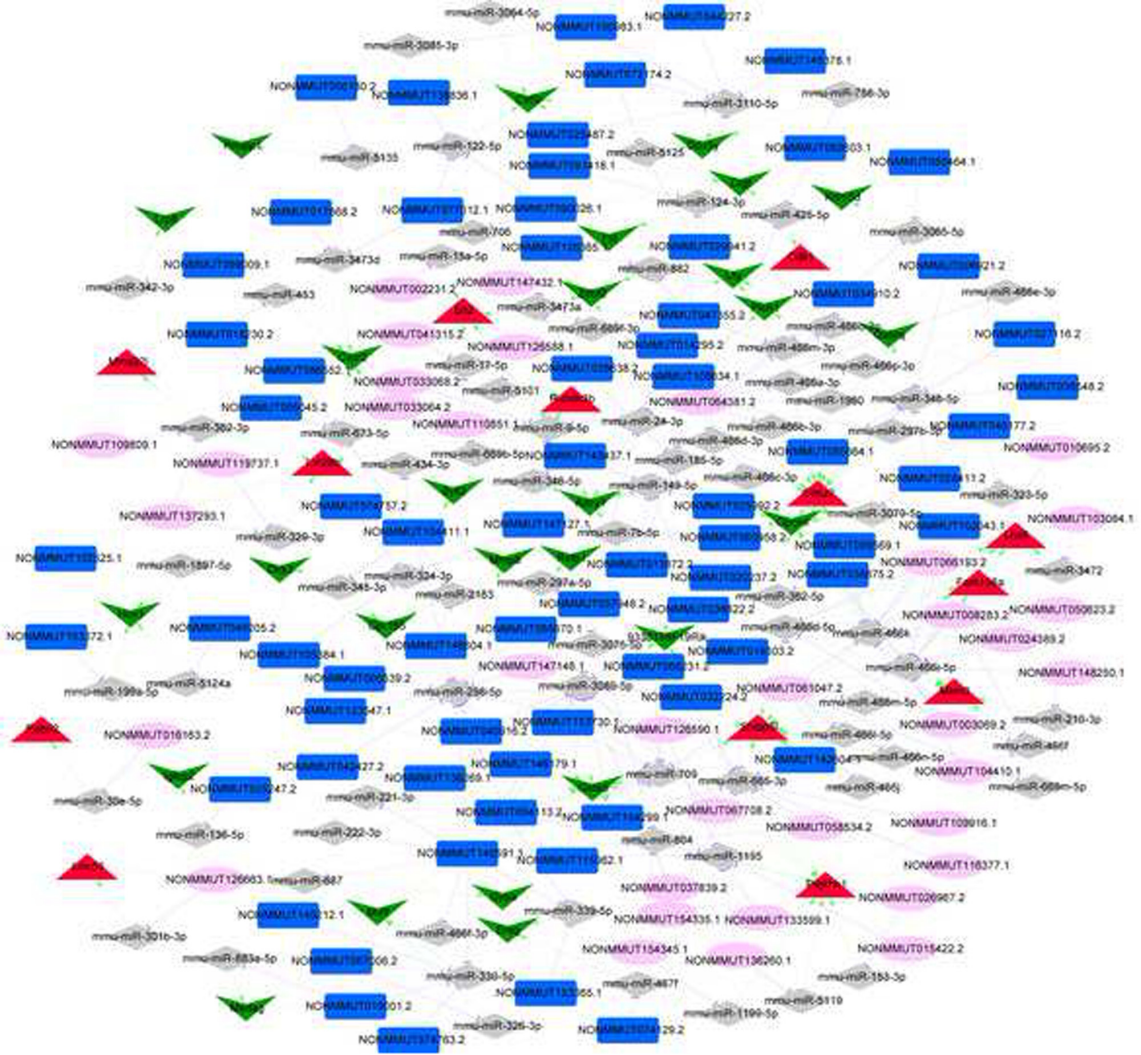
ceRNA network (red triangle represents upregulated mRNA; green inverted triangle represents downregulated mRNA; gray rhomboid represents miRNA; dark blue rectangle represents downregulated lncRNAs; pink ellipses represent upregulated lncRNAs; the green arrow represents the miRNA–mRNA regulatory relationship; the purple T-line represents the lncRNA–miRNA regulatory relationship; the yellow line represents the lncRNA–mRNA co-expression relationship).

A total of 396 miRNA–mRNA regulatory pairs were predicted for mRNAs in the lncRNA–mRNA co-expression relationships. Additionally, 54,916 lncRNA–miRNA pairs were predicted. The lncRNA–miRNA–mRNA pairs were then screened. Combining with the positive co-expression relationship between mRNA and lncRNA (correlation coefficient >0.8), 1,575 lncRNA–miRNA–mRNA pairs were obtained, including 349 lncRNAs, 150 miRNAs, and 68 mRNAs. Due to the large number of nodes, we focused on screening the lncRNA–miRNA–mRNA pairs with a positive co-expression correlation coefficient of >0.95 of mRNA–lncRNA, and constructed the ceRNA network, as shown in Figure 7. The network contained 129 regulatory pairs of miRNA–mRNA, 206 regulatory pairs of lncRNA–miRNA, and 134 co-expression pairs of lncRNA–mRNA. The hub nodes (top 5) in the network are shown in Table 1. NONMMUT140591.1 had the highest degree in the ceRNA network and was involved in the ceRNA of NONMMUT140591.1-mmu-miR-298-5p-*Gata5* (GATA binding protein 5).

**Table 1:** Node connectivity degree ranking in ceRNA network (top 5)

Node	Degree	Type
NONMMUT140591.1	15	lncRNA_Down
NONMMUT064381.2	15	lncRNA_Up
NONMMUT061047.2	11	lncRNA_Up
NONMMUT047355.2	9	lncRNA_Down
NONMMUT057948.2	8	lncRNA_Down
Gata5	19	mRNA_Down
Opcml	18	mRNA_Down
Plekhh1	18	mRNA_Up
Ccna2	15	mRNA_Up
Stxbp5l	14	mRNA_Up
mmu-miR-466i-5p	17	miRNA
mmu-miR-298-5p	16	miRNA
mmu-miR-466k	15	miRNA
mmu-miR-665-3p	12	miRNA
mmu-miR-709	10	miRNA

## 4 Discussion

In this study, urea accumulation by UT-B deletion results in the aberrant expression of 588 lncRNAs and 194 mRNAs. These DEMs were significantly enriched in the inflammation-related pathway. A lncRNA–mRNA co-expression network and a ceRNA network were built based on the DEMs and DELs. C7 (complement C7)–NONMMUT137216.1 co-expression pair had the highest correlation coefficient. NONMMUT140591.1 had the highest degree in the ceRNA network and was involved in the ceRNA of NONMMUT140591.1-mmu-miR-298-5p-*Gata5* (GATA binding protein 5).

This study identified more downregulated genes than upregulated genes. The downregulated mRNAs were significantly involved in the IL-17 signaling pathway, cytokine–cytokine receptor interaction, and the chemokine signaling pathway. Interestingly, these pathways are inflammation related. Studies have reported that chronic inflammatory diseases have come into focus as a risk factor for the development of cardiovascular dysfunction [19,20]. Previous studies have focused more on the fact that urea exacerbates the progression of the disease or plays a role in certain complications. It has also been reported that high urea have toxicity in other cells and tissues. Urea induces the expression of proapoptotic proteins in human aortic endothelial cells [21], resulting in increased mitochondrial reactive oxygen species (ROS) production and activation of pro-inflammatory pathways which deteriorates the quality of life of patients with chronic kidney disease [22]. A previous study reported that congenital complete heart block is considered as an inflammatory process in patients with a structurally normal heart, which is due to transplacental transfer of maternal autoantibodies [23]. Clinical evidence suggests that tissue injury in both acute kidney injury and heart failure has immune-mediated inflammatory consequences that can initiate remote organ dysfunction [24]. The cardiovascular system is the main route of urea transportation. Therefore, urea specifically transmits organ crosstalk information from the kidney to the heart. The urea level is very stable in almost all conditions, which makes urea a reliable crosstalk language that only the kidney speaks. Wang et al. found that urea should be considered as an independent factor causing disease directly, and urea toxicity plays an important role at the cellular and systemic level [25]. Urea perseprobably participates in the pathogenesis of cardiovascular disease, insulin resistance, intestinal disease, and contributes to an overall accelerated ageing phenotype [26]. Our study may further support the report above. We speculated that urea accumulation by UT-B deletion may promote cardiac conduction block via the inflammatory process.

Co-expression analysis has been highly successful in the function of revealing genes, and has contributed greatly to the understanding of gene regulation systems [27–29]. Our study constructed a lncRNA–mRNA co-expression network. The C7–NONMMUT137216.1 co-expression pair had the highest correlation coefficient. C7 is a component of the terminal complement cascade [30]. The complement system is part of the humoral innate immune response, which forms a cascade of more than 30 proteins bound to the target surface or present in the plasma [31]. It has been suggested that the complement system plays a key role in the inflammatory response after acute myocardial infarction [32]. However, no one has reported that it has a role in cardiac conduction block to the best of our knowledge. As we mentioned above, cardiac conduction block may be associated with the inflammatory process. So, we speculated that urea accumulation by UT-B deletion may promote the co-expression of C7 and NONMMUT137216.1 to participate in the progression of cardiac conduction block.

NONMMUT140591.1 was a downregulated lncRNA in UT-B knockout mice. Additionally, it had the highest degree in the ceRNA network and was involved in the ceRNA of NONMMUT140591.1-mmu-miR-298-5p*-Gata5*. Gata5 belongs to the zinc finger transcription factor GATA family (GATA1–6), which is abundantly expressed in various endoderm- and mesoderm-derived tissues, predominantly in the embryonic heart [33]. Gata5, having partially overlapped function with Gata4 and Gata6, has been reported to have an important role in cardiovascular development [34]. Previous studies have suggested that Gata5 is associated with the bicuspid aortic valve [35,36]. Given the important role of Gata5 in cardiovascular development, we speculated that UT-B knockout may be involved in the cardiac conduction block trough downregulation of NONMMUT140591.1 as a ceRNA to regulated Gata5 expression.

However, this study had several limitations. First, the sample size of this study is relatively small. A large sample size is needed. In addition, the results identified from bioinformatics analyses have to be verified through *in vitro* and *in vivo* experiments.

## 5 Conclusion

To conclude, we found that urea accumulation by UT-B deletion may promote cardiac conduction block via inflammatory process. The ceRNA NONMMUT140591.1-mmu-miR-298-5p*-Gata5* may be a potential molecular mechanism of UT-B knockout-induced cardiac conduction block.
